# The Nature and Manipulation of Dental Amalgams and a Standardized Amalgam Technic

**Published:** 1922-04

**Authors:** Robert K. Brown

**Affiliations:** Ann Arbor, Mich.


### THE NATURE AND MANIPULATION OF DENTAL AMALGAMS AND A STANDARDIZED AMALGAM TECHNIC

ROBERT K. BROWN, D.D.S., ANN ARBOR, MICH.

An amalgam is a combination of two or more metals, one of which is mercury. An alloy is a union of two or more metals. Hence an amalgam is an alloy containing mercury.

The production of a dental amalgam depends on the property of mercury of dissolving most other metals to the point of saturation, forming alloys that set or harden when allowed to stand for a time. The resulting amalgam is probably due to the formation of a chemical compound between mercury and one or more of the constituent metals, and also probably from a mechanical mixture to some extent.

Dental amalgams are divided into two classes, viz:

Class I. High percentage silver alloys whose general formulae are,

| | |
|---|---|
| Silver | 65-68% |
| Tin | 26-28% |
| Copper | 3-5% |
| Zinc | ½-2% |

This class of alloys is free from decrease in volume, stronger, more stable in form, works harder and sets quicker than those in Class II.

Class II. Low percentage silver alloys whose general formulae are,

| | |
|---|---|
| Silver | 43-48% |
| Tin | 48-58% |
| Zinc | 1-2% |

These alloys are weaker, lighter in color, easily amalgamated and slower setting than those in Class I.

The high silver alloys reach their maximum strength in about ten days and remains stationary. The low silver alloys reach their peak in five days, although it gradually increases with age.

The high silver alloys are about seventy-five per cent. stronger than the low silver and due to their greater affinity for mercury for the same weight of alloy produce fillings about twenty-five per cent. greater in volume than the low silver alloys. This is to be considered when purchasing an alloy from an economical standpoint.

Solutions and mixtures generally possess the properties of their constituents and this is true of amalgams to a great extent. Silver and tin being the basis of the alloy we would expect their properties to predominate. Zinc and copper are added as they possess certain qualities which are desirable to use as modifiers.

We will briefly enumerate the different properties possessed by the metals used in amalgams generally.

#### PROPERTIES OF SILVER

1. It unites with mercury in all proportions.
2. It controls the setting of the mass.
3. It increases volume change.
4. It increases edge strength.
5. It lessens flow.
6. It tarnishes in the mouth.

#### PROPERTIES OF TIN

1. It unites with mercury in all proportions at all temperatures.
2. It forms a weak crystalline compound.
3. It retards setting.
4. It decreases in volume.
5. It increases the flow.
6. It imparts plasticity to the mass.

It is seen that silver and tin are diametrically opposed in a great many of their properties and are essential to an amalgam if proportioned correctly.

PROPERTIES OF COPPER

1. It unites with mercury with difficulty at ordinary temperatures.

2. A definite proportion hastens setting.

3. It increases edge strength.

4. It lessens flow.

5. It does not change in volume or tarnish in the mouth.

PROPERTIES OF ZINC

1. It unites with mercury easily and in definite proportions.

2. It increases volume.

3. It hastens setting.

4. It increases edge strength.

5. It lessens flow, improves the color and imparts smoothness to the mix.

Gold as far as present research has developed, imparts no desirable qualities and several undesirable ones, such as springiness when packing and toughness when used in an alloy.

It is possible some properties of gold may later be discovered making it a more desirable and valuable addition.

Certain points controlling the behavior of alloys other than amalgam are well known to metallurgists and these also control the behavior of an amalgam. These are packing or casting pressure, packing time, trituration or mixing time, size of the alloy used, the temperature at which amalgam is kept, its annealing and its age.

It has been observed that there is a contraction immediately after the combination of alloy and mercury in the mass, then a slow expansion. This is followed by a slower contraction bringing the volume back to approximately that at which the alloy was first made, provided the alloy is of high percentage silver class and properly made.

However, pressure in packing will modify this greatly due to increase in action between the mercury and the alloy for combining substances brought into more intimate contact speed the reaction. A continual pressure will also result in a similar condition.

Trituration, or mixing of mercury and alloy, has to do with the contact of the alloy and mercury and will accelerate the reaction, although the ratio of alloy and mercury used is a great factor and must be considered closely with it. Varying percentages of alloy and mercury are found to effect an amalgam's behavior very greatly if not properly controlled.

The finer the alloy particles the more acceleration there is to the reaction and the earlier the appearance of characteristic features.

The question of temperature is not under the control of the dentist and though important, can not be considered.

Every day in our practice we see constantly before us the result of the phenomena of contraction and expansion in alloys. This accompanies the setting of amalgam. The variance in volume change is under the control of the manufacturer to quite an extent. He can modify it by his composition of the alloy, or its annealing. As a result, the manufacturers now produce amalgams from a fixed formula as they can follow a definite technic in production and secure as pure raw material, that is, containing the same percentages of impurities in different batches, as they wish. This was impossible a few years ago.

In the annealing of an alloy, temperature is the main factor. The amount of temperature necessary to anneal an alloy varies with different alloys. 120 degrees F., for two to seven days will generally do it, or the alloy may be suspended in a test tube in boiling water (212 degrees F.) for twenty minutes. Annealing affects the strength, volume change, rate of setting and the percentage of mercury necessary to make a plastic mass in an amalgam.

This is used as a trade getter by the manufacturer, for by his various methods of annealing or not annealing at all, he produces his slow, medium and quick setting alloys.

Low temperature and a longer time bring about a more complete annealing due to a restoration of the molecules of the alloy to their original position.

As we stated, annealing seems to increase the strength of high silver alloys up to a certain point. Manufacturers now anneal their product to produce the desired properties after they have annealed them. Summarizing the result of annealing an alloy we may say it affects the volume change, reducing expansion in those that expand, and increasing contraction in those that contract. It increases the strength of the high silver alloys, slows the rate of setting and requires less mercury to amalgamate.

The strength of amalgams is considered in regard to their crushing resistance and their resistance to flow. Crushing resistance is the property of an alloy to resist force without fracturing. It is studied as the properties of the metals used to form the alloys individually, and the properties of the amalgam mass.

Copper and tin give strength, while tin decreases it and zinc gives strength in a relation between that of copper and silver. Hence the composition of the alloy, then the process of annealing, chilling and alloying are important factors in measuring its efficiency. An amalgam's strength will be increased with a use of greater packing pressure.

The temperature of an amalgam when subjected to stress has a marked influence on its ability to resist it. This is due to the fact that the alloy is worked in the cold state and decreases in strength with a rise in temperature more markedly than an alloy that has been cast and allowed to cool.

The alloy must have been triturated three to five minutes depending on the speed of the operator, remembering that if the maximum time is used, the packing must be done quickly. Enough mercury must always be used to react fully with the alloy. Too little mercury will give a weak mass as some alloys will not be dissolved in the mercury.

An amalgam's strength depends also on the age of the filling. For the low packing pressures we used in the more inaccessible points in the mouth, a greater time, say three or four months should elapse before the maximum strength has developed in the filling.

A freshly cut alloy will not allow of a complete union of alloy and mercury on account of the rapidity of the reaction. Hence proper annealing will give proper time to triturate and increase the product's strength even in a freshly cut alloy.

Flow is the property of an alloy to resist force without change in shape. Tin has the property of continued flow under pressure, while silver and copper will flow and stop until a greater pressure is applied. Flow is also modified by the percentage of mercury in the amalgam, its manner of trituration, the condition of the cut alloy, and the manipulation of the mass.

All washing or annealing of alloys should be left to the manufacturer unless the alloy has been contaminated. To wash an alloy small quantities of hydrochloric acid or alcohol are used.

Amalgam is placed near gold in its thermal conductivity. It is insoluble in the mouth unless having a high copper content. Copper and tin seem to give them some antiseptic properties as we can all attest.

An operator should buy high silver alloys and only from a reputable manufacturer. Fillings are the best form to use. The slow setting or the annealed form is bought if the manufacturer offers a quick and slow setting form.

The essentials for a standardized amalgam technic may be listed as follows:

1. Correct cavity preparation.

2. The use of a reputable high silver alloy.

3. The adaption of a well made matrix if the cavity does not possess four walls.

4. The use of the rubber dam where possible.

5. Correct trituration of the mass.

6. Correct instrumentation and condensation of the amalgam.

7. The restoration of proper contour, contact and a high polish to the restoration being made.

Going briefly into detail, cavities are prepared essentially as those for foil, except that the cavo surface angles should be made wider to increase the strength of the filling, all connections between the occlusal and any of the four walls of the tooth should be as wide as possible to protect against flow, enamel walls should be beveled the depth of the enamel and all possile means of retention utilized.

We have considered the alloy to be used previously.

A matrix for each individual case in hand should be made. We use 36 gauge sheet copper. This is annealed by heating to a red heat over a flame and plunged into water, repeating this two or three times. The metal is now sterilized as well as workable. This band is fitted to the tooth, in mesio-occlusal or disto-occlusal fillings it does not have the ends soldered to each other giving us a complete matrix around the tooth, but is used as it is, cutting it about one-eighth of an inch beyond the cavity margin. It is now contoured to the case, being sure it covers the cervical floor. It is marked on the inside where the contact should be if the contact is to be restored and a hole is drilled through the band with a No. 4 round bur. Thus when the band is held in position on the tooth by ligatures, the amalgam will be forced through this hole and intimate contact with the adjacent tooth secured. In order not to have this contact present a duplication of this hole, it is thinned away around all its edges on the inner or cavity side with a small Miller stone until the band slopes into the contact hole, and not with a sharp angle as would otherwise be.

At each cervical end the band is turned up at the corner to engage the ligature we use. This is waxed ligature thread. A pair of shears are used to cut from the contact hole we have made in the band to its cervical edge. Thus when the amalgam has set sufficiently, we cut the ligature from the matrix and pull each end up and around the contact we have established and do not disturb it as the slit we made weakened the matrix to this point and allows of its easy removal.

Use a double ligature and a surgeon's knot to hold matrix in position and place the matrix in disto and mesio occlusal cavities after the dam is applied.

Cotton, spunk or orangewood wedges may be used at the cervical to hold the matrix close to the cervical floor. They are placed between the adjacent tooth and matrix in the embrasure and sufficient pressure applied to bring the matrix close to the cervical floor of the cavity. This prevents an overhanging cervical on the filling with its very undesirable sequela.

When a restoration is made involving nearly all the greater portion of the coronal part of the tooth, a circular copper band is made by soldering the ends together to a previous measurement of the tooth by means of a dentimeter. This band is contoured so all cervical margins are covered, the contact points are marked by a burnisher in their proper location on the inner surface of the band and cut with a bur. These are slit to the gingival as before. The bands are trimmed so the patient closes his mouth normally as this matrix must stay in position for twenty-four hours at least. Lugs are turned up in the cervical area of the band at points where cavity margins are not present, these hold the ligature down in place. This band is ligated as the other, the cervical floor burnished in and held by suitable means then the dam is applied.

Have all condensing instruments on hand, put the alloy and mercury, correctly proportioned in a mortar and triturate for about two or three minutes. The mass is then removed to the hand, the excess mercury removed during hand manipulation until the mass can be rolled into a rope without breaking and will show thumb markings plainly. Excess mercury should not be squeezed out in muslin or chamois as this causes a loss of too much time.

Flat or cup shaped instruments with serrated ends should be used for packing as the W. G. Crandall set of sixteen. Seven of these are bayonet shaped for use in the upper jaw and are of different sizes, and seven are binangles for use in the lower jaw. Two are used to brush off the excess mercury that comes to the surface during condensation.

In packing, heavy steady pressure is best. Wedge the mass against cavity walls, then in the center as this wedges against the walls and secures closer adaptation. Use as large plugges as possible, and do not break up the mass any more than necessary in packing. Be sure and fill all undercuts or crevices thoroughly. The amalgam mass must not be too sloppy but rather stiff to secure a good margin. Fill the cavity to overflowing, then condense with a mallet and orangewood stick. Allow it to harden somewhat. Remove the dam and proceed with the carving. Use the set of Frahms or Hollenbacks carvers designed for this purpose. Carve from the amalgam to the margins. With the dam off, you can carve to correct occlusion and articulation. Here the artistic ability of the dentist can be brought out.

Simple matrixes as for M. O. and D. O. cavities can now be removed and the excess trimmed off and the cervical finished with Black's amalgam knives. This is far more easily done now than after the amalgam has set and is very essential.

Fine sandpaper strips are passed below the contact point and the proximal surface roughly polished. At a subsequent setting at least forty-eight hours later the final polish is given. Strips, stones and discs are used, then a mirror finish given with pumice on felt or bristle wheels and whiting. At yearly intervals these fillings should be inspected, any expansion of the mass ground down and the filling repolished.

We should not use amalgam in the six anterior teeth either in the upper or lower jaw and seldom anterior to the molars. Its greatest value perhaps is in the restoration of badly decayed and broken down teeth. Here it is more often indicated than crowning, for if correctly done will cause much less subsequent irritation.

In devital teeth, posts of twelve to sixteen gauge nickel silver wire may be cemented in the canals, nickel for added retention both on the part of the cement and of the amalgam and the pulp chamber squared out for added retention.

In vital teeth small gold or indio-platinum posts may be cemented in areas away from the pulp as added retention.

The dental profession at large is doing very poor amalgam work. Cheap alloys are used, their manipulation not understood, or if known, not applied, and no consideration given to the application of a proper matrix, the securing of a contact point or points as needed, no carving attempted and polishing never thought of.

From one of our most invaluable filling materials we have secured results that stare each of us daily in the face. We see the flat, unpolished surfaces, the lacking contact, those wide, overhanging cervicals, and those cavity forms where retention seems to be merely a trust in God.

To remedy this we must educate our patients so as to secure a higher fee for this class of work. Our cavity preparation is as exacting as that of the gold foil or inlay, its correct insertion almost as difficult, and its polish if anything, harder.

We should consider amalgam on a basis with gold work. It is not an easy working mass, to plug a hole which has never been carefully prepared with all traces of decay removed. It is indicated in many places, and in some of our practices is used more than any other material. It has done us good service in the past, even badly as we have mistreated it. What it would do if used as our present knowledge indicates is conjecture. I have an idea it would be raised far above the plane it now occupies. Let us all give it a fighting chance.

